# Polyphenols Targeting Oxidative Stress in Spinal Cord Injury: Current Status and Future Vision

**DOI:** 10.1155/2022/8741787

**Published:** 2022-08-22

**Authors:** Fahadul Islam, Sristy Bepary, Mohamed H. Nafady, Md. Rezaul Islam, Talha Bin Emran, Sharifa Sultana, Md. Amdadul Huq, Saikat Mitra, Hitesh Chopra, Rohit Sharma, Sherouk Hussein Sweilam, Mayeen Uddin Khandaker, Abubakr M. Idris

**Affiliations:** ^1^Department of Pharmacy, Faculty of Allied Health Sciences, Daffodil International University, Dhaka 1207, Bangladesh; ^2^Faculty of Applied Health Science Technology, Misr University for Science and Technology, Giza, Egypt; ^3^Department of Pharmacy, BGC Trust University Bangladesh, Chittagong 4381, Bangladesh; ^4^Department of Food and Nutrition, Chung Ang University, Anseong-Si, Gyeonggi-Do 17546, Republic of Korea; ^5^Department of Pharmacy, Faculty of Pharmacy, University of Dhaka, Dhaka 1000, Bangladesh; ^6^Chitkara College of Pharmacy, Chitkara University, Punjab 140401, India; ^7^Department of Rasashastra and Bhaishajya Kalpana, Faculty of Ayurveda, Institute of Medical Sciences, Banaras Hindu University, Varanasi, Uttar Pradesh 221005, India; ^8^Department of Pharmacognosy, College of Pharmacy, Prince Sattam Bin Abdulaziz University, Al-Kharj 11942, Saudi Arabia; ^9^Department of Pharmacognosy, Faculty of Pharmacy, Egyptian Russian University, Cairo-Suez Road, Badr City 11829, Egypt; ^10^Centre for Applied Physics and Radiation Technologies, School of Engineering and Technology, Sunway University, 47500 Bandar Sunway, Selangor, Malaysia; ^11^Department of Chemistry, College of Science, King Khalid University, Abha 62529, Saudi Arabia; ^12^Research Center for Advanced Materials Science (RCAMS), King Khalid University, Abha 62529, Saudi Arabia

## Abstract

A spinal cord injury (SCI) occurs when the spinal cord is deteriorated or traumatized, leading to motor and sensory functions lost even totally or partially. An imbalance within the generation of reactive oxygen species and antioxidant defense levels results in oxidative stress (OS) and neuroinflammation. After SCI, OS and occurring pathways of inflammations are significant strenuous drivers of cross-linked dysregulated pathways. It emphasizes the significance of multitarget therapy in combating SCI consequences. Polyphenols, which are secondary metabolites originating from plants, have the promise to be used as alternative therapeutic agents to treat SCI. Secondary metabolites have activity on neuroinflammatory, neuronal OS, and extrinsic axonal dysregulated pathways during the early stages of SCI. Experimental and clinical investigations have noted the possible importance of phenolic compounds as important phytochemicals in moderating upstream dysregulated OS/inflammatory signaling mediators and axonal regeneration's extrinsic pathways after the SCI probable significance of phenolic compounds as important phytochemicals in mediating upstream dysregulated OS/inflammatory signaling mediators. Furthermore, combining polyphenols could be a way to lessen the effects of SCI.

## 1. Introduction

Neurodegenerative disorders (NDDs) progressively affect millions worldwide as significant causes of disability and death, despite progress in considering various dysregulated routes in the pathophysiology of NDDs. The main pathophysiological processes of NDDs are still unknown [1–4]. Spinal cord injury (SCI) is an NDD that causes sensory-motor impairment and significantly lowers the standard of living. SCI is becoming more common among people aged 14.6 to 67.6 years old, and men are four times more likely than women [5, 6]. SCI has primary and secondary phases from a pathophysiological standpoint. The secondary step comprises inherent oxidative stress (OS), autophagic, apoptotic, and inflammatory routes. Direct injuries occur after spinal mechanical trauma [7].

In contrast, extrinsic routes have an essential role in SCI, such as glial scar development and destruction [8]. Extrinsic pathways are coupled with intrinsic processes such as OS, neuroinflammation, and neuroapoptosis (e.g., axonal signaling). Thus, the preceding pathogenic pathways negatively affect neurodegeneration and neurodegenerative mechanisms, eventually leading to apoptosis. Antioxidant defenses modulate neuroinflammatory and neuroapoptosis responses, which influence microglia, astrocytes, and related mediators and have a considerable position in the initiation and development of SCI [9, 10].

It is crucial to highlight that developing new plant medications has a compelling track record in producing unconventional therapeutics. Incidentally, the plant kingdom has demonstrated encouraging outcomes in that against SCI. Polyphenols/phenolic combinations are obtainable phytochemicals and can act as multiple targeted drugs with excellent selectivity and minimal toxicity among natural substances, because of their broad biological activity and therapeutic properties are now used in contemporary medications to construct and acquire novel treatments. In many NDDs, these substances have been regarded as reliable nutritional mediators with potent repressive impacts on OS and inflammation [11]. Emerging research has recently focused on utilizing organic neuroprotective polyphenols with putative antioxidant properties to treat SCI and NDDs [12]. This review discussed about the oxidative-mediated polyphenols' role in controlling and managing SCI.

## 2. Methodology

**Figure 1:**
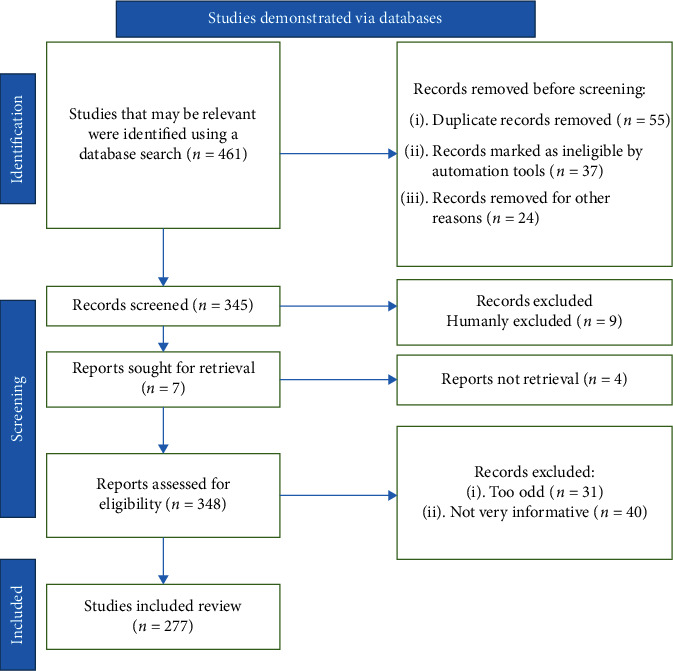
The stages of picking data for inclusion in the existing research are illustrated in a flow chart; *n* = number of literature reports.

PubMed, Scopus, and Web of Science were all used to conduct this literature review. The terms polyphenols, SCI, oxidative stress, reactive oxygen species, preclinical studies, and clinical studies were utilized. We selected and analyzed English-published research papers, narrative review articles, and primary research articles until June 2022. An algorithm used the flowchart imposed in Figure 1 (according to Page et al.'s guidelines [13]) and contained all of the steps/selection constraints for the required literature.

## 3. Spinal Cord Injury Pathophysiology

**Figure 2:**
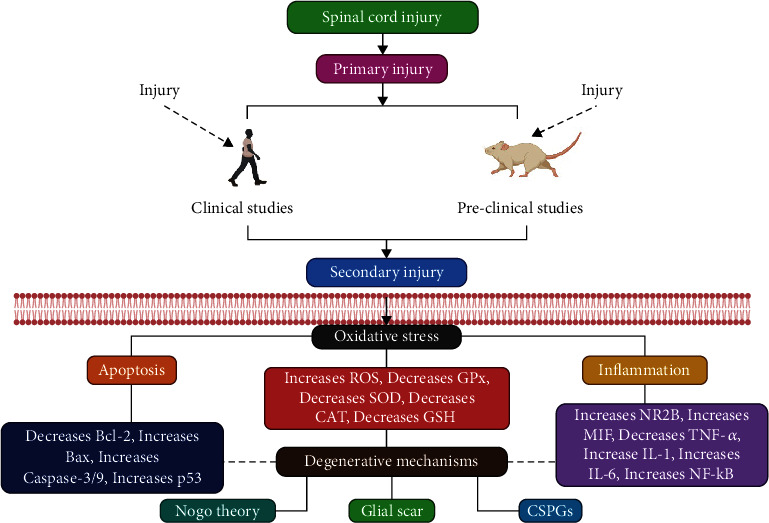
Pathophysiology of SCI: spinal cord injury. ROS: reactive oxygen species; GPx: glutathione peroxidase; SOD: superoxide dismutase; CAT: catalase; GSH: glutathione; MIF: macrophage migration inhibitory factor; TNF-*α*: tumor necrosis factor-alpha; and NF-*κ*B: nuclear factor kappa B.

SCI is categorized into primary, secondary, and chronic [14, 15]. The first stage is the physical forces related to the original traumatic event, often the essential factors of injury severity, causing the first stage. Compression, shearing, laceration, and severe stretch/distraction are examples of these forces [16]. Following the original injury, a series of subsequent occurrences occur. The damage worsens in the second stage, and neurological impairments and consequences worsen [17, 18]. After the first injury, secondary SCI is a gradual and progressive injury (Figure 2).

Furthermore, a chronic stage could affect the orthograde and retrograde routes and brian-specific regions; moreover, according to the time scale, chronic stages can start from days to years following the damage [19, 20]. Several vascular alterations are detected during the secondary cascade [21]—neutrophils and macrophages and role in releasing superoxide anion and hydrogen peroxide to sanitize the wounded area. Nicotinamide adenine dinucleotide phosphate (NADPH) oxidase is a significant superoxide anion originator of superoxide anion that plays a role in activating the hematogenous phagocytic cells [22]. Moreover, the phagocytic inflammatory cells work as reactive oxygen species (ROS) producers. At the same time, the free radicals respond to polyunsaturated fatty acids, which lead to a phospholipid structural design disruption of cellular and subcellular organelle membranes. Furthermore, aldehyde molecules produced by lipid peroxidation prevent metabolic enzymes, such as Na^+^/K^+^-ATPase, from working precisely [23].

SCI causes an increase in cytokines containing tumor necrosis factor-alpha (TNF-*α*), interleukin-1 (IL-1), and interleukin-6 (IL-6), as well as overexpression of nuclear factor kappa B (NF-*κ*B), activator protein 1 (AP-1), c-Jun N-terminal Kinase (JNK), and other inflammatory and apoptotic factors like p38, mitogen-activated protein kinase (MAPK), and prostaglandin E2 (PGE2) [24]. The generation of excitation amino acids involving glutamate from damaged cells increases the discharge of excitation amino acids after SCI [25, 26].

Additionally, the glial scar formation, microglia/macrophages, reactive astrocytes, and extracellular matrix molecules—particularly chondroitin sulfate proteoglycans—at the chronic phase play a vital part in preventing axon growth by acting as a protective border [27–29]. Therefore, developing reliable methods and treatments for SCI patients becomes imperative. Reduced ROS levels are an essential approach for SCI management, which can be accomplished by employing antioxidants or drugs that standardize or modulate ROS signaling routes [30, 31].

## 4. Spinal Cord Injury and Oxidative Stress

**Figure 3:**
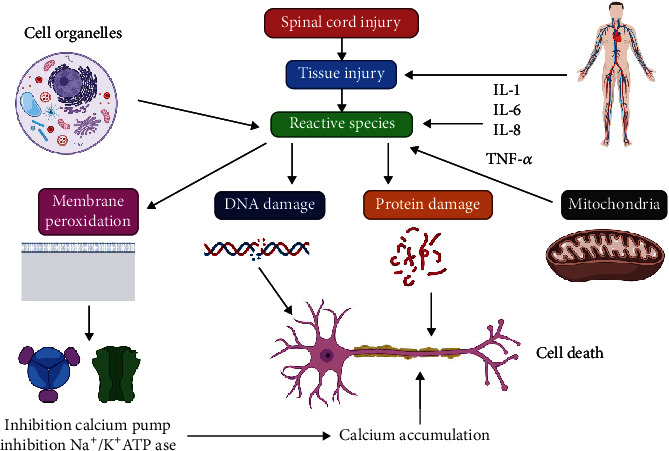
SCI can be facilitated by oxidative stress. TNF-*α*: tumor necrosis factor-alpha.

Reactive nitrogen species (RNS) and ROS are frequently formed endogenously. However, an increased ROS construction may outpace the antioxidant defense capability, leading to OS and oxidative destruction (Figure 3) [32–35]. Superoxide is created by the NADPH oxidase (NOX), mitochondrial electron transport chain, and xanthine oxidase (XO), which response to nitric oxide (NO) manufactured by the nitric oxide synthase (NOS) to generate the peroxynitrite (ONOO) [36, 37].

Superoxide dismutase (SOD) is an enzyme that transforms oxygen (O_2_) into hydrogen peroxide (H_2_O_2_). There are two similar forms of SOD: (1) copper (Cu)/zinc(Zn)-SOD and (2) manganese(Mn)-SOD. Zn plays a considerable part in the antioxidant defense scheme. According to the databases, the Zn condition and time-dependent modifications following SCI are still unknown [38–42]. The analysis of Zn dynamics in 38 cervically damaged SCI patients yielded a prediction prototype for continuing functional prediction [41]. Heller and colleagues [42] looked at the vigorous variations in serum Zn intensity in short periods throughout the preliminary 72 hours after injury to see a link between early changes in total Zn serum levels and NDDs and patient outcomes. They discovered that patients with the cognitive disease have higher median Zn concentrations in the initial 9 hours after injury than patients with vertebral fractures who do not have neurological dysfunction. They established that the result is associated with early Zn level dynamics and could be an investigative tool for these patients. Alterations in serum Zn levels allow early assessing the risk of neurological damage [42].

In this context, it was discovered that Zn therapy aided motor control restoration in the 28 days that followed SCI and reduced ROS and increased antioxidant potential [43]. The Fenton reaction allows H_2_O_2_ to produce the highly reactive hydroxyl radical (HO•), that considers the leading cause of lipid peroxidation in the presence of iron. Catalase (CAT) and glutathione peroxidase (GPx) convert H_2_O_2_ to water and oxygen [44]. SOD, CAT, GPX, and glutathione reductase are the primary endogenous antioxidant enzymes [34].

The enzyme GPX is selenium (Se) dependent. By neutralizing reactive oxygen species (ROS) via GPX and reversible oxidation to glutathione disulfide, GSH acts as an antioxidant (GSSG). Glutathione reductase transforms into GSH. Meanwhile, XO produces superoxide but catalyzes the conversion of xanthine to UA, a compound that may scavenge superoxide. HO is the primary antioxidant in biological fluids. In rats, Se nanoparticles were shown to treat OS-induced SCI [45]. According to Seelig et al., Cu and Se concentrations upon intake and Se and ceruloplasmin levels after one day were indications of likely SCI clearance [46]. Within the secondary injury stage, magnesium (Mg) is assumed to play an important role. A better probability of neurological recovery has been associated with reduced Mg serum concentrations during the first seven days [47]. Mg acts by blocking ROS generation and lipid peroxidation precisely [48].

Acrolein, a reactive aldehyde generated endogenously by lipid peroxidation and involved in SCI, is more responsive than the other HNEs and causes glutathione deprivation [49]. To investigate the antioxidant potential of SCI patients, Bastani et al. examined a vast scope of antioxidant and OS markers. When evaluating persons with SCI to controls, they observed that urine F2-IsoP and specific enzymes (NOX and XO) in vastus lateralis biopsies enhanced while SOD decreased [50, 51].

## 5. Polyphenols in Spinal Cord Injury

**Figure 4:**
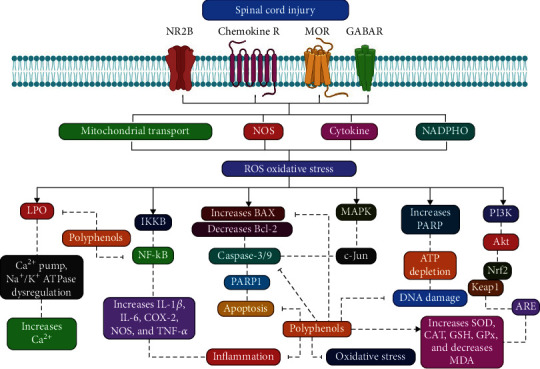
Action mechanism illustration of polyphenols blocking spinal cord injury. LPO: lactoperoxidase; TNF-*α*: tumor necrosis factor-alpha; NF-*κ*B: nuclear factor kaa-B; GPx: glutathione peroxidase; SOD: superoxide dismutase; CAT: catalase; GSH: glutathione; COX-2: cyclooxygenase-2; MDA: malondialdehyde; Nrf2: nuclear factor erythroid 2–related factor 2; PARP1: poly-ADP ribose polymerase 1 (PARP-1).

To reduce OS after SCI, many natural polyphenolic combinations have been used [52]. These compounds impede the restoration of molecules following free radical damage and control various dysregulated pathways/mediators, such as blocking production. OH. Such polyphenols have formerly been prospective neuroprotective therapeutics in other OS-related NDDs (Figure 4) [53–55].

### 5.1. Epigallocatechin Gallate

**Figure 5:**
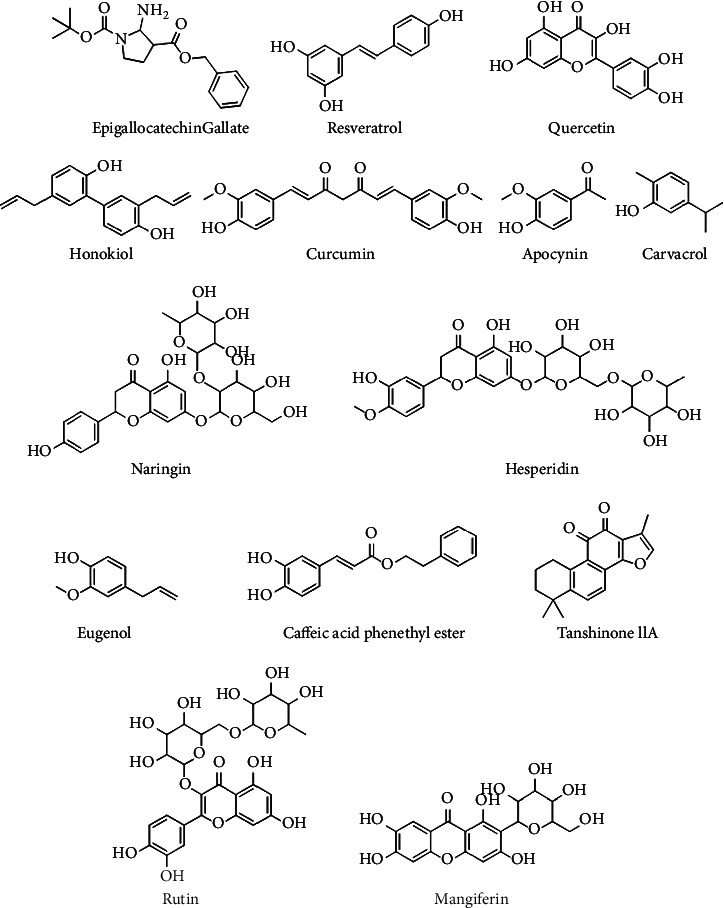
Chemical configurations of some efficient chemical complexes as opposed to spinal cord injury.

The primary compound of tea catechins is epigallocatechin gallate (EGCG) (Figure 5), often called epicatechin. This composition is related to the biological functions of green tea extracts [56]. EGCG's anti-apoptotic, anti-inflammatory, and antioxidant actions have been demonstrated to prevent against NDDs [57], brain injury [58], SCI [59], and peripheral nerve damage [60] in many experiments conducted. The hydroxyl groups in the catechins ring B and D cause them to interact with free radicals [61]. For 24 hours, various doses of green tea polyphenols (Table 1) (50–200 *μ*g/mL) prevented spinal neurons from oxidative damage caused by H_2_O_2_ [62].

In vitro experiments revealed that PC12 cells to 0-2000 mol/L of EGCG hindered ROS generation [63]. Dosages of EGCG (10, 25, or 50 mg/kg, i.p.) drastically diminished NADPH/neuronal nitric oxide synthase (nNOS) representation following nerve damage in mice [53] and inhibited neurodegeneration by activating the cyclic adenosine monophosphate (cAMP) for 18 days with 25-75 mg/kg dosage scale of myeloperoxidase (MPO) function, inducible TNF-*α*, interleukin 1 beta (IL-1*β*), poly-ADP ribose polymerase (PARP), nitric oxide synthase (iNOS), and cyclooxygenase-2 (COX-2) representation were all reduced in the rat spinal cord after a 50 mg/kg dose of EGCG [60, 64].

Khalatbary et al. also swiftly exhibited a 50 mg/kg i.p. injection of EGCG and 1 hour after SCI lowered malonaldehyde (MDA) [65]. In a rat spinal cord organic culture, EGCG at a five-molar level for 48 hours suppressed OS and preserved motor neurons, according to *in vitro* experiments [66]. Thermal hyperalgesia was minimized in mice after administering 30 mg/kg of EGCG for a week following SCI, inhibiting the expression of RhoA and TNF-*α* [67].

### 5.2. Resveratrol

Resveratrol (3,4′,5-trihydroxystilbene) (Figure 5) is a natural phytoalexin identified in *Veratrum grandiflorum*, grape, and peanut that protects counter to stress damage fungal growth [68, 69]. Resveratrol is a potent antioxidant because it scavenges free radicals, protects against ROS-stimulated DNA damage [70], and reduces the generation of H_2_O_2_. Resveratrol significantly suppressed oxidized glutathione reductase [63], GSH function, TNF-*α*, and IL-1*β* production [64]. Additionally, resveratrol promoted autophagy by stimulating the nuclear factor erythroid 2–related factor 2 (Nrf2) gene and prevented programmed cell death by increased expression of the sirtuin 1 (SIRT1) gene [71, 72].

According to some studies, resveratrol is a SIRT1 activator that may prevent OS, inflammation, and apoptotic neurons, according to some studies [73]. The SIRT1/Akt1 pathway was developed by resveratrol, resulting in cell survival [74]. Suppressing the TLR-4/MyD88/NF-*κ*B enhanced mitochondrial function/biogenesis [75].

By surpassing the NF-*κ*B signaling pathway, the resveratrol might reduce the SCI health consequence severity [76]. Resveratrol (Table 1) (100 mg/kg, i.p.) induced the activity of p-AMPK, Bcl-2, and SIRT1, while lowering the transcription of p62, caspase-3, caspase-9, and Bax, which following SCI. Resveratrol was also reported to protect neurons by downregulating via the SIRT1/AMPK signaling pathway [77, 78].

Apoptosis-related genes were revealed to be helpful in the SCI rat model by Liu et al. [79]. Resveratrol exhibited anti-apoptotic impacts after SCI, according to Zhang et al., by reducing associated p53, caspase-3, and cytochrome C [80]. Additionally, resveratrol suppressed neuroinflammation following SCI by triggering autophagy by the AMPK/mTOR pathway [81]. Resveratrol significantly benefited neuronal autophagic flux to minimize programmed cell death and stimulate operational repair in rats to post to SCI [82].

A further study demonstrated that resveratrol (200 mg/kg) diminished programmed cell death, OS, and inflammation [30]. In mice, a particular quantity of resveratrol improved autophagic proteins while reducing apoptotic ones [83]. Senturk et al. reported that resveratrol (Table 1) (10 mg/kg) exhibited anti-inflammatory characteristics after SCI [84]. Polydatin (20, 40 mg/kg), a glucoside of resveratrol [85], via the Nrf2/heme oxygenase-1 (HO-1) pathway, suppressed OS and protected apoptosis post-SCI [86].

### 5.3. Quercetin

Flavonoids such as Quercetin (Figure 5) are observed in several fruits, vegetables, and grains. It exhibits anti-inflammatory, anti-carcinogenic, antioxidant, and antiviral activities, among other pharmacological attributes. Quercetin has also been demonstrated to enhance neuronal dysregulation and mental/physical malfunction by inhibiting lipid peroxidation and capillary penetrability and encouraging mitochondrial biogenesis [87–90]. Quercetin's phenolic hydroxyl groups can effectively scavenge. OH, superoxide anions, and LPO [91]. Quercetin can also connect to conversion metals and inhibit oxidation and decrease, forming metal chelates that can be used to neutralize transition metals, notably copper and iron [92]. Quercetin's neuroprotective properties have been widely exhibited in several in vivo studies. After brain damage considerably reduced GSH levels and MPO function [93]. In traumatic brain damage [94], quercetin boosted the activities of SOD, GPx, and AT, lowered the increased MMP-9 level [95], and regulated the tropomyosin receptor kinase B (TrkB) and brain-derived neurotrophic factor (BDNF) [96].

Quercetin (Table 1) (30 mg/kg) also reduced OS, spinal cytokine secretion, and glial cell facilitation of GFAP [97]. Additional studies revealed that a ten-day i.p. quercetin management at a 20 mg/kg/day dosage scale could mitigate monosodium Glu-induced neurotoxicity by lowering p38MAPK, decreasing OS, and boosting GFAP transcription [98]. According to Azevedo et al. [89], quercetin (25, 50, and 100 mg/kg) mitigated OS-induced degeneration by lowering LPO, which was in agreement with Liu et al. [99, 100].

Following SCI, a 7-day i.p. processing of 20 mg/kg quercetin inhibits the p38MAPK/iNOS signaling pathway and synchronizes secondary OS by blocking the BDNF and JAK2/STAT3 signaling pathways [101]. Quercetin administration at a frequency of 0.25 mol/kg diminished MPO expression, according to Schültke et al. [102]. In addition, a particular dose of quercetin provided during three days of SCI enhanced overall antioxidant levels while lowering NO and MDA levels [103]. Quercetin raised overall antioxidant potential and paraoxonase function in rats following SCI [104].

A further research paper discovered that delivering 20 mg/kg of quercetin could safeguard against SCI-stimulated OS by behaving as an antioxidant and anti-inflammatory [105]. Wang et al. observed that quercetin (50 mol/kg) attenuated proinflammatory cytokines while elevating anti-inflammatory cytokines relevant to oxidative mechanisms. The treatment significantly attenuated the cystic cavity size while enhancing macrophage polarization, neuronal function, and axonal survival [106]. Based on in vivo and in vitro investigations, quercetin (7.5 mg/kg) suppressed oligodendrocyte necroptosis after SCI by modulating the STAT1 and NF-*κ*B pathways [107]. Jiang et al. discovered that 100 mg/kg of quercetin lowered ROS construction, IL-1, TNF-*α*, and IL-18 in female rats following SCI [108]. Therefore, quercetin appears to be a favorable treatment for reducing OS after neurodegeneration and SCI.

### 5.4. Honokiol

*Magnolia grandiflora* has a pleiotropic lignan called honokiol (Figure 5) [109]. Antioxidant [110], anti-inflammatory [111], analgesic [112], depressive [113], antitumorigenic [114], and neuroprotective [115] actions are among its therapeutic benefits. Honokiol has been shown to reduce OS factors in tissue diversity, involving the heart [116], liver [117], kidney [118], and brain [119]. Honokiol reduced ROS generation in microglial cells via the ERK/NADPH oxidase pathway [120]. To exhibit neuroprotective effects, it also triggered Nrf2 [121], suppressed xanthine oxidase (XO), and regulated the PI3K/Akt pathway [122]. Furthermore, honokiol protected mitochondrial respiratory chain enzymes by targeting PKC, MAPKs, and NF-*κ*B [123–125]. 20 mg/kg of honokiol decreased the generation of proinflammatory cytokines and prevented neutrophil permeation and microglial stimulation in a rat version of SCI, all of which are linked to oxidative factors [126]. In ischemic brains, 10 g/kg of honokiol reduced neutrophil infiltration and ROS production while maintaining Na^+^/K^+^-ATPase function and mitochondrial biogenesis against OS [113]. Honokiol also conserved mitochondrial respiratory chain enzyme [125]. In a rat model of SCI, 20 mg/kg of honokiol lowered the manufacture of proinflammatory cytokines, blocked neutrophil penetration, and prevented microglial activation, all associated with oxidative factors [126]. 10 g/kg of honokiol (Table 1) reduced neutrophil infiltration and ROS generation in ischemic brains while maintaining Na^+^/K^+^-ATPase activity and mitochondrial biogenesis [113].

### 5.5. Curcumin

Curcumin (Figure 5) is an organic polyphenol substance isolated from the *Curcuma longa* rhizome [127, 128]. In many studies, curcumin has antioxidant, anti-inflammatory, and anticancer estates, which have antioxidant, anti-inflammatory, and anticancer properties. Curcumin exerts anti-inflammatory actions via upregulating the PPAR- linked with the NF-*κ*B pathway [129, 130]. Curcumin inhibited the stimulation of NF-*κ*B, lowered the production of COX-2, IL-1, IL-6, IL-8, and TNF-*α* [131], and boosted the SOD activity [132]. Curcumin's anti-inflammatory impact after SCI has been linked to suppression of NF-*κ*B, IL-1*β*, IL-6, and TNF-*α* activity, as well as an enhancement in Nrf2 [133] and stimulation of the TLR4/NF-*κ*B signaling route [134].

Curcumin generated antioxidative preservation via Nrf2 routes and a reduction in ROS as a consequence of NF-*κ*B stimulation [135]. In treating SCI, curcumin also affects the mTOR signaling pathway [136]. Curcumin, a more potent antioxidant that targets antioxidant enzymes such as GPx and SOD than vitamin E, has been reduced by methoxy and phenolic groups [137]. Curcumin elevated the CDGSH iron sulfur domain 2 (CISD2) as a durability gene due to its activities in Ca^2+^ metabolism after SCI. CISD2 improved BCL-2/Beclin-1 binding. It is guarded against programmed cell death and mitochondrial dysfunction. At the ER stress, CISD2 reduced a rise in excitotoxic Ca^2+^ [138].

Curcumin reduced neuron death and inhibited neuronal death following SCI, according to Lin et al. [139]. In the long-term treatment of SCI, curcumin outperformed methylprednisolone by lowering Bax and caspase-3 while increasing Bcl-2 [140]. Following curcumin therapy in humans or mice, tetrahydrocurcumin is among the most common curcumin metabolites isolated from the liver cytoplasm and small intestine [141]. In SCI patients, tetrahydrocurcumin (80 mg/kg/day) has been reported to lower OS and death [142]. Curcumin decreased inflammatory cytokines with pro-apoptotic effects in rats after SCI [143].

Curcumin entirely inhibited TGF-*β* following SCI. They also discovered that curcumin inhibits NF-*κ*B, a protein implicated in the apoptotic and inflammatory mechanisms [144]. Curcumin's anti-apoptotic action was also exhibited in the spinal cord damage rat model, later being given intravenously. Curcumin was also found to decline caspase-3 [145], enhance Bcl-2 [146], and have anti-inflammatory antioxidant estates [147]. In a rabbit model of SCI, curcumin was discovered to block apoptotic (caspase-3) [147].

### 5.6. Naringin

Naringin (Figure 5) is considered a flavanone glycoside attained from citrus fruits. Naringinase hydrolyzes it to yield naringenin, which can effortlessly intersect the blood-brain barrier [148]. The inflammatory and OS reactions in adults' brains were controlled by naringin therapy. Naringin also has neuroprotective estates by stimulating neurotrophic factors and constraining apoptosis [149, 150]. Naringin can be an apoptotic inhibitor because the inflammatory factors and apoptotic mediators are linked. Following SCI, naringin (Table 1) (20, 40 mg/kg, p.o.) raised BDNF and vascular endothelial growth factor (VEGF) levels while inhibiting brain apoptosis [151]. BDNF reduced apoptosis and MAPK pathways via interacting with TrkB [152, 153], although the *β*-catenin/GSK-3*β* signaling route has been found to promote remyelination following SCI [154]. Naringenin, a naringin aglycone analog, has shown promising neuroprotective benefits and may be used in SCI in the future. Naringenin diminished the expression of IL-6, TNF-*α*, and CXCL10 mRNA in the spinal cord, which is an essential factor in apoptosis [155].

### 5.7. Apocynin

Apocynin (Figure 5), also known as acetovanillone, is an organic polyphenolic substance extracted from the rhizomes of *Apocynum androsaemifolium* [183]. Apocynin is a nicotinamide adenine dinucleotide phosphate (NADPH) oxidase inhibitor that suppresses p47phox's serine phosphorylation and prevents it from binding to gp91phox, delaying NADPH oxidase activity [184]. H_2_O_2_ and myeloperoxidase (MPO) stimulate apocynin, resulting in the formation of an apocynin radical. NADPH oxidase is inhibited by thiol-oxidizing compounds [185], a significant source of ROS in the cell [186]. This method has significantly altered redox-sensitive signaling pathways in neuroinflammation in different NDDs, particularly SCI. Sun and colleagues have found that apocynin (50 mg/kg) (Table 1) reduced SCI-induced neurodegenerative in rats by diminishing inflammatory cytokine production, improving glutathione (GSH)/SOD activity, and decreasing MPO and malondialdehyde levels (MDA). Apocynin (5 mg/kg) inhibited apoptosis after SCI by lowering FasL stimulation and phospho-JNK, P38, inflammatory cytokines (IL-1, TNF-*α*), and NF-*κ*B representation levels [171]. Corresponding to research by Liu et al., apocynin can aid histology results and forelimb motor control restoration following SCI. Furthermore, Zhang and coworkers demonstrated the prospective neuroprotective estates of apocynin by decreasing neuroinflammation in spinal cord injured rats by suppressing the growth of NADPH oxidase-mediated ROS [172]. In an SCI chronic animal experiment, ROS and lipid peroxidation were similarly reduced by apocynin, implying an indirect control of apoptosis [169].

### 5.8. Carvacrol

Carvacrol (Figure 5) is a monoterpenoid phenolic product of cymene and has been demonstrated to have anxiolytic [187], depressive [188], antibacterial, antioxidant [189], anticancer, antimutagenic [190], anti-inflammatory [191], and antihepatotoxic properties [192]. Carvacrol strengthened the regulations of Nrf2 and ERK1 in PC12 cells that had been suppressed by cadmium [193]. Cells following exposure to iron ions and in cells exposed to H_2_O_2_ exhibited anti-carcinogenic characteristics via HO-1 [194, 195]. The Fenton reaction combines an excess of iron ions with oxygen, causing oxidative damage such as mitochondrial dysfunction and LPO [19]. Carvacrol also has anti-inflammatory and proinflammatory cytokine modulating properties [196]. After administering (25, 75, and 150 mg/kg), it inhibited OS factors like MDA, GSH, and NO [173]. However, more investigations are required to identify the neuroprotective properties of carvacrol following SCI via oxidative mechanisms.

### 5.9. Hesperidin

Hesperidin (Figure 5) is an anti-inflammatory, antioxidant, anticancer, and anti-apoptotic flavanoglycone obtained from citrus fruits [197, 198]. Hesperidin regulated Nrf2/ARE/HO 1 and TGF1/Smad3 signaling, which decreased OS and inflammation [199]. Hesperidin modulation of the ERK/MAPK pathway is implicated in the production of HO-1 and Nrf2 in an *in vitro* investigation based on OS [200]. *In vitro*, hesperidin triggered Nrf2/ARE/HO-1 and upregulated the Keap1-Nrf2/HO-1 pathway, enhancing the action of antioxidant enzymes in kidney tissue [201]. As a result of stimulating the Nrf2/HO-1/ARE and PPAR mechanisms, it reduced OS and inflammation [201, 202].

### 5.10. Rutin

The flavonol glycoside rutin, commonly identified as vitamin P, is derived from buckwheat [203]. Rutin (Figure 5) has a number of pharmacological properties, such as cytoprotection, antioxidant [204], anticancer [205], vasoprotection [206], neuroprotective effects [207], and anti-inflammation [163]. Rutin lowered OS by increasing CAT function, decreasing LPO and protein carbonyl content, and modulating the MAPK [208] and iNOS/ Nrf2 signaling pathways. In ischemic neuronal apoptosis, rutin suppressed LPO and p53 expression, enhanced antioxidant defense enzymes, and lowered ROS generation [209]. In mice, it alleviated diabetic neuropathy by lowering OS via HO-1 and Nrf2 [210]. Rutin boosted the transcription of BDNF, CREB, and ERK1 genes in the hippocampus at 100 mg/kg [211] and shielded PC12 cells against sodium nitroprusside stimulation by regulating the PI3K/Akt/mTOR and ERK1/2 pathways [212]. Oral medication with 10 mg/kg rutin for three weeks reduced OS [213].

A further study noticed that three-day rutin (Table 1) (50 and 100 mg/kg) substantially reduced ROS, MDA, IL-1, IL-18, and TNF-*α* [163]. Rutin protected cells from OS and apoptosis caused by H2O2 in vitro studies by directing the Bax/Bcl-2 ratio and the NF-*κ*B/p65 signaling route, managing ROS, reducing LPO, and maintaining the intracellular antioxidant enzyme activities [214]. Rutin also safeguarded neurons from oxidative DNA damage and degeneration resulting from a lack of food [215]. Furthermore, 30 mg/kg rutin in the SCI animal paired with mild hypothermia for three days after SCI decreased inflammatory factors by blocking the TGF-*β*/Smad route [215].

### 5.11. Mangiferin

Mangiferin (Figure 5) is a bioactive xanthonoid extracted from various mango components. It is a potent antioxidant [216] with a variety of health benefits, notably immunomodulatory [217], antiviral [218], anti-inflammatory [219], antidiabetic [220], anticancer [221], and analgesic [222] activities. Mangiferin inhibits LPO and DNA damage by neutralizing free radicals and generating mangiferin-iron complexes [216, 223]. In an *in vivo* study, mice were recovered from cadmium chloride contamination by administering 50 mol/L of mangiferin for 4 hours, which reduced LPO rates and increased GSH, CAT, GST, and SOD activity [224]. Mangiferin increased Nrf2 levels, altered NQO1 expression, and increased ROS levels *in vitro* research [225]. Interestingly, 20 and 100 mg/kg of mangiferin triggered the Nrf2/HO-1 pathway in a dose-dependent approach in a brain injury model [177]. Mangiferin (Table 1) (20 and 100 mg/kg) for 30 days after SCI significantly decreased MDA at the same time as significantly boosted SOD, CAT, and GPx [178]. Mangiferin's neuroprotective properties in concentrations of 10, 25, and 50 mg/kg 30 days following SCI were connected with diminished spinal cord edema, reduction of OS, and inflammatory condition [226].

### 5.12. Caffeic Acid Phenethyl Ester

Honeybee propolis contains phenethyl caffeate [227]. Because of the associated hydroxyl groups in the catechol ring, it has antioxidant [228], anti-inflammatory [229], antibacterial [230], anticancer, and cytotoxic effects [231]. The phenethyl ester of caffeic acid inhibits NF-*κ*B [232] and protein tyrosine kinase [233]. Hypoxic-ischemic brain injury models inhibit lipoxygenase activity [234] and limit calcium-induced cytochrome c release [235]. Following ischemia-reperfusion injury, caffeic acid phenethyl ester suppressed superoxide anion generation and XO [236] and decreased MPO and Na^+^/K^+^ ATPase capacities [237]. Caffeine's phenethyl ester increased HO-1 synthesis by activating Nrf2 and the extracellular signal-regulated kinases (ERK) signaling route [238]. It binds to Keap1, allowing Nrf2 to better connect to ARE [239]. MDA, LPO, and total oxidant action were reduced after SCI with an intrathecal infusion of 1 g/kg caffeic acid phenethyl ester. After SCI, it boosted antioxidative mediators [240], even as it decreased IL-6 levels in tissue and serum [241]. In a similar vein, Ak et al. found that caffeic acid phenethyl ester (10 g/kg) infusions lowered TNF-*α* and IL-1*β* levels after SCI [179].

After SCI, 10 mol/kg of this phytochemical enhanced motor function and decreased lesion size by lowering IL-1*β*, NOS, and COX-2 expression [180]. Caffeic acid phenethyl ester, 10 mol/kg (Table 1), was given before surgery to minimize ischemic damage in the spinal cord and to enhance microcirculation by blocking endothelial cell lysis by activated leukocyte proteases [242]. It also inhibited ROS and iNOS catalytic performance at a 50 mol/mL dosage, which had neuroinflammatory effects [243].

### 5.13. Tanshinone IIA

Tanshinone IIA (Figure 5) is extracted from the roots of *Salvia miltiorrhiza*. Tanshinone IIA has been found to have anti-apoptotic and anti-inflammatory properties in investigations [244]. Tanshinone IIA's antioxidant development is associated with efficient communication among DNA and lipid peroxidation product avoidance, DNA conservation by inhibiting NADPH oxidase, lipid peroxidation, and lipid-free radical clearance [245, 246]. Tanshinone IIA also inhibited the onset of neuroinflammation in neurodegenerative pathologies by preventing the production [247]. MAPKs are also critical signaling mediators that control cell development and death [248]. Tanshinone IIA (20, 50 mg/kg) (Table 1) has been demonstrated to suppress inflammation and apoptosis during SCI by decreasing NF-*κ*B, MAPK, IL-1*β*, TNF-*α*, IL-6, iNOS, and caspase-3 boosting Bcl-2 [181]. Other investigations [249, 250] determined the spinal levels of inflammatory factors after tanshinone IIA treatment. These inflammatory factors also interact with apoptotic factors, as aforementioned. Tanshinone IIA has been shown to have the ability to improve neuronal autophagic factors and pathways (PI3K/Akt/mTOR) [251].

### 5.14. Eugenol

Eugenol (Figure 5), often known as clove oil (4-allyl-2-methoxy phenol), is an organic chemical derived from the *Syzygium aromaticum* (clove) plant [252]. Eugenol has antitumor [253], anti-microbial [254], anti-inflammatory [255], and antioxidant properties. It has been proven that proinflammatory cytokines, inflammation enzymes, and antioxidative enzymes reduce inflammation [256]. Eugenol has been shown to have therapeutic efficacy by lowering TRPV1 and sodium channels [257], connecting with Ca^2+^ channels [258], and boosting autophagy via the AMPK/mTOR pathway [259]. Eugenol lowered OS, inflammatory markers, and caspase-3 [182]. In neuroprotective effects, Eugenol increased Bcl-2 but decreased Bax [238] and TNF-*α* [239]. It has also been demonstrated to stimulate neuronal autophagy by the Akt/AMPK route [259].

## 6. Clinical Studies

Polyphenols are potential secondary metabolites with a comprehensive scale of favorable health outcomes. The US Food and Drug Administration (FDA) has acknowledged curcuminoids as relatively reliable and highly allowed effective forms in clinical studies, even at concentrations of up to 12,000 mg/day [260]. In controlled clinical research, curcumin's impacts on inflammatory and stress markers in 100 osteoarthritis patients of both genders have been investigated [261]. In a prospective randomized open-end blinded examination (PROBE) of 80 individuals with knee osteoarthritis, researchers discovered that consuming 30 mg of curcumin three times a day (p.o.) for four weeks decreased COX-2 concentrations [262]. Another RDBPC analysis [263] shows the anti-inflammatory efficacy of oral curcumin (400mg/3 times a day, p.o.) in type 2 diabetic cases and a substantial decrease in MDA, IL-6, and TNF-*α* levels.

In one hundred individuals with SCI, curcumin was significantly connected to decrease osteoporosis development and bone metabolism markers after six months [264]. According to randomized, parallel-group outcomes controlled clinical research on 20 participants, the InflanNox tablet (curcumin 1200 mg/day) has additional anti-inflammatory and antioxidant characteristics, lowers IL-1*β*, and improves depression and anxiety in SCI patients [265]. In 50 individuals with multiple sclerosis, administration of nanocurcumin (80 mg/day) was linked to a considerable increase in TGF-*β* and IL-10 expression [266]. Nanocurcumin was governed in a randomized of 40 diabetes people. In this investigation, nanocurcumin was discovered to be an antioxidant that may minimize OS and free radicals [267].

Polyphenol supplements (200mL/day) reportedly regulated plasma homocysteine concentrations in 48 Alzheimer's patients in an eight-month multiple center RDBC experiment [268]. In a multicenter, double-blind clinical investigation, thirty-four diabetic patients with neuropathy (aged 21 to 72) were given a topical preparation including quercetin to reduce OS [269]. Verlaet et al. showed antioxidant properties in a randomized controlled experiment examining the treatment properties of the herbal, polyphenol-rich extract [270]. Furthermore, another study found that meals high in polyphenols could increase cognitive reserve [271]. Another polyphenol-rich extract has shown promising antioxidative consequences in healthful people and those suffering from NDDs [272–274].

## 7. Conclusion and Future Perspectives

The complicated pathophysiological mechanisms in SCI seem to be orchestrated by OS to influence other interrelated pathways, such as neuroinflammation. Thus, an interaction between OS and neuroinflammatory/apoptotic pathways is complex.

In this line, Nrf2/Keap1/ARE, SOD, CAT, GSH, MDA, HO-1, and XO have significantly reduced the associated pathways/mediators contributing to neuroprotection in NDDs and SCI. Because of the polyphenol's shortcomings, researchers must apply novel drug delivery strategies in clinical studies, such as nanoformulations. Nanoformulations of polyphenols are proposed to overcome such restrictions due to the management indicated above and the advantageous effect of nanoparticles in boosting spinal cord medication distribution. It will enable the chemical's favorable impacts on SCI and other NDDs. To address SCI difficulties, metal nanoparticles (iron oxide, gold, silver, and so on), liposomes, and inorganics have all been utilized to create nanoparticles [275].

Equivalent recommendations will aid in raising understanding of the complexities of dysregulated signal transduction pathways after the SCI and the significance of discovering new and more effective multitarget alternative natural intermediaries with more excellent safety and efficacy among the scientific community. The exact molecular pathogenesis and signaling pathways associated with NDDs and the secondary phase of SCI must be revealed in further research studies. The mediators represent promising options to prevent associated pathogenicity in an oxidative way. Polyphenols are suggested to be the primary focus in this line of work as alternatives to interventions with fewer complications and greater efficacy.

Polyphenols/phenolic compounds are secondary metabolites with a broad scale of biological activity and health improvements exploited in modern medication to generate novel drugs [276]. Clinical studies are currently evaluating the therapeutic effect of polyphenols in the treatment of NDDs; however, clinical research to investigate the promise of polyphenols in treating following SCI consequences is lacking [277]. Therefore, well-designed clinical trials will aid in revealing polyphenols' therapeutic promise in addressing sensory-motor dysfunction after SCI and pave the way to address any recommendations for the future of their administration. The role of OS in modifying the inflammatory and apoptotic pathways in NDDs, with a particular focus on SCI, was investigated in this work. As potential multitarget neuroprotective treatments, we also emphasized the need to synthesize polyphenols and phenolic compounds that proinflammatory cytokines, extrinsic axonal related pathways, and other pathways involved with OS. Co-administering polyphenols/phenolic chemicals may also help treat SCI side effects. These research projects will explore potential pharmacological targets for avoiding, controlling, and treating NDDs and SCI.

## Figures and Tables

**Table 1:** Various preclinical investigations have investigated the effect of polyphenols in combating OS and in the after of SCI.

Polyphenol	Dose/concentration	Study model	Pharmacological mode of actions	References
Epigallocatechin gallate	50 mg/kg (i.p), instantly and one h after SCI	Female SD rats	Diminished Bax and MDA; improved Bcl-2	[65]
	30 mg/kg (i.p.); 7 days after SCI	Female BALB/c mice	Decreased TNF-*α* and RhoA	[67]
	10, 20 mg/kg (i.t)	Female SD rats	Decreased Bax; increased Bcl-2 and BDNF	[156]
	25 mg/kg (p.o), 1 and 6 h later to SCI	Male adult CD1 mice	Decreased Bax, TNF-*α*, MPO, MDA, NF-*κ*B, iNOS, PARP; increased Bcl-2	[157]

Resveratrol	1 and 10 mg/kg (p.o); 30 min earlier to SCI	Wistar male rats	Decreased NO and MDA	[159]
	400 mg/kg (p.o.); 10 days after SCI	SD male rats	Decreased MDA and IL-6	
	50, 100 mg/kg (i.p.)	SD male and female rats	Decreased MDA; improved Na^+^, K^+^-ATPase activities	[160]
	200 mg/kg (i.p.); until three days after SCI	SD rats	Decreased MDA, MPO, IL-1*β*, IL-10, and TNF-*α*; increased SOD	[79]
	50, 100, 200 mg/kg (i.v.); until seven days after SCI	Female mice	Decreased p38MAPK; NF-*κ*B	[158]
	100 mg/kg (i.p.)	Long Evans female rats	Decreased MDA, NO, and TBARS	[161]
	200 mg/kg (i.p.); directly after SCI	Wistar male rats	Enhanced SOD, GPx, and CAT	[162]
	100 mg/kg (i.p), directly after SCI	Male SD rats	Diminished TNF-*α*, IL-1*β*, IL-10, and mTOR; enhanced AMPK, LC3, and Beclin-1	[81]
	200 mg/kg (i.p), Immediately after SCI	Male C57BL/6 mice	Decreased Bax; increased Bcl-2, LC3, and Beclin-1	[83]

Quercetin	10,100mg/kg (i.p), first 3 days after SCI	Wistar male rats	Decreased MDA and NO	[103]
	100 mg/kg (i.p.) for three days following SCI	Male SD rats	Decreased ROS, IL-1*β*, IL-18, and TNF-*α*	[108]
	20 mg/kg (i.p.), twice per day for seven days following SCI	Wistar albino rats	Decreased MDA, IL-6, TNF-*α*, and caspase-3	[105]
	Up until ten days following SCI, 7.5 mg/kg (i.p.), two times per day	Female SD rats	Decreased TNF-*α*, iNOS, NF-*κ*B, and IL 12; enhanced IL-4 IL-10, and TGF-*β*	[107]

Honokiol	20 mg/kg (i.p.)	Female SD rats	Decreased MPO, iNOS, COX-2, IL-1*β*, IL-6, and TNF-*α*	[126]
	50, 100 mg/kg (i.p.), until three days following SCI	Female SD mice	Decreased MDA, ROS, and TNF-*α*	[163]

Curcumin	100 mg/kg (i.p), 15 min following SCI	Male SD rats,	Decreased IL6, IL1*β*, TNF-*α*, NF-*κ*B, and TLR4	[164]
	200 mg/kg (i.p), 1week before SCI	Male Wistar albino rats	Degraded caspase-3, IL1*β*, TNF-*α*, MDA, SOD, and GSH	[145]
	60 mg/kg (i.t), directly after SCI, until three weeks, once weekly	Wistar rats	Decreased IL4, IL1*β*, IL12, and TNF-*α*,	[143]
	200 mg/kg (i.m), until eight weeks after SCI	Male SD rats	Decreased caspase-3, Bax, and Bcl-2	[140]
	60 mg/kg (i.m), 30 min after SCI, until three weeks	Male SD rats	Decreased mTOR, p62, and Akt	[165]

Naringin	50, 100 mg/kg (p.o.), three days before SCI until seven days after SCI	Male SD rats	Diminished TNF-*α*, IL8, IL-1*β*, and IL-6	[166]
	20 mg/kg (i.p.), directly and one h after SCI	Female SD rats	Reduced MDA and Bax; enhanced Bcl-2 and GSH	[167]
	50, 100 mg/kg (i.p), 1week before SCI	Female SD rats	Decreased TNF-*α*, IL-1*β*, IL-6, NF-*κ*B, MPO, MDA, and SOD; increased GSH, and CAT	[168]
	20, 40 mg/kg (p.o), until six weeks after SCI	Female SD rats	Decreased caspase-3 and Bax; increased Bcl-2 and BDNF	[151]

Apocynin	0.1 mg/kg (i.t)	Male SD rats,	Decreased ROS	[169]
	100 mg/kg (i.p)	Male SD rats	Decreased Caspase-1, ROS, NF-*κ*B, JNK, and p38	[170]
	5 mg/kg (i.p), 1 and 6 h after SCI	Male CD1 mice	Decreased NADPH oxidase, JNK, p38, FasL, MPO, and Bcl-2	[171]
	5 mg/kg (i.p), 1 and 6 h after SCI until 1week	C57BL/6 female mice	Decreased ROS	[172]

Carvacrol	25,75 and 150 mg/kg (i.p)	Male SD rats	Diminished TNF-*α*, IL-1*β*, MPO, and NF-*κ*B	[173]

Hesperidin	100 mg/kg; 7 days before SCI until seven days after SCI	Female SD rats	Decreased IL-1*β*, NF-*κ*B, and PARP; increased SOD, HO-1, and p-p38	[174]

Rutin	30 mg/kg (i.p.)	Rats	Diminished MDA; IL-6; TNF-*α*; and NF-*κ*B; increased SOD; GSH; CAT	[175]
	30 mg/kg (i.p.), until 3 days	Male SD rats	Decreased TNF-*α*; MDA; ROS; TGF-*β*1; and Smad2	[176]

Mangiferin	20, 40 mg/kg (i.p.), until 30 days after SCI	Male SD rats	Decreased MDA, NF-*κ*B; increased SOD, GPx, and CAT	[177]
	10, 25, 50 mg/kg (i.p.)	SD rats	Decreased MDA, NF-*κ*B, TNF-*α*, and caspase-9; increased CAT, SOD, and GSH	[178]
	0.2 mg/kg (i.p.), 1 h after SCI	Male SD rats	Decreased iNOS, p38MAPK, MDA, and SOD	[92]
	0.25 *μ*mol/kg (i.p.), 1 h after SCI	Wistar male rats	Decreased MPO	[102]

Caffeic acid phenethyl ester	10 *μ*L; 1 *μ*g/kg (i.t.), 1 h after SCI	Wistar female mice	Decreased MDA, SOD, and TOA; increased TAC	[179]
	10 *μ*g/kg (i.p.), 30 min after SCI	Wistar female rats	Increased IL-1*β*, and TNF-*α*	[180]

Tanshinone IIA	50 mg/kg (i.p) 1h before SCI (20 mg/kg) until 7 days after SCI	Male SD rats	Decreased TNF-*α*, NF-*κ*B, MAPK, and JNK	[181]

Eugenol	25, 50 mg/kg (p.o), until seven weeks after SCI	Female SD rats	Decreased, NF-*κ*B, and iNOS; increased SOD, and CAT	[182]

## Data Availability

All data used to establish the conclusions of this study are integrated into the article.
